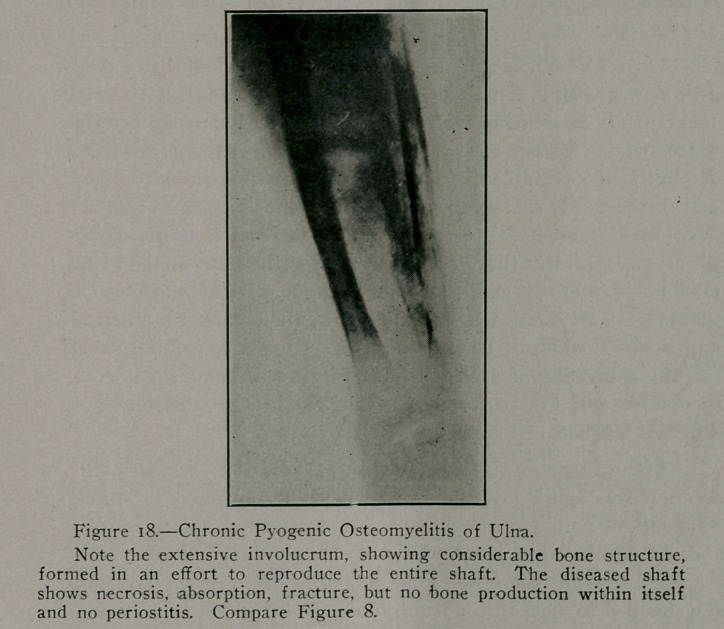# Selections and Abstracts

**Published:** 1913-05

**Authors:** 


### SELECTIONS AND ABSTRACTS

### THE SERUM DIAGNOSIS OF PREGNANCY.

One of the most important and useful diagnostic measures resulting from serological study is that discovered recently for the determination of pregnancy. The new test devised by Abderhalden is based on the ferment action of blood serum. It has been observed that protein substances injected into the blood ofdogs and rabbits cause the development of proteolytic ferments within the serum. Even cane sugar injected into dogs, intravenously, will cause an appearance of invertin in the blood serum. This may appear as early as fifteen minutes after the injection. In other words, the introduction of proteins, carbohydrates, and fats directly into the circulation calls forth enzymes, which denaturize the foreign bodies by a process of digestive disintegration. Similarly, the escape into the maternal circulation of cells or other products of the placenta, gives rise to enzyme production within the maternal serum. The new test depends upon the determination of the digestive action of blood from pregnant women on placental protein.

Petri, in the Zentralblatt fur Gynakologie, (No. 7, Feb. 15, 1913), discusses Abderhalden's discovery and its value in early pregnancy during the first half, and believes the serum test of Abderhalden removes the difficulty. This test indicates the presence of a proteolytic enzyme within the blood serum of the mother. This proteolytic enzyme acts upon the potein derived from the placenta. It is believed that cells from the chorionic villi, and even from the trophoblast produce enzymes within the maternal blood. Schmol has shown the presence of syncytial elements in the pulmonic vessels in a case of eclampsia The production of enzyme is protective in character. There are two methods for perforating Abderhalden's test:

Method 1. A five per cent. solution of the placenta in distilled water is prepared. One cubic centimetre of serum from a pregnant woman is mixed with one cubic centimetre of the placental solution in a test-tube. The test-tube is incubated at body temperature. The tube is then examined with the polariscope. The breaking down of the protein molecules will cause an increased rotation of rays of polarized light.

Method 2. The placetna after being washed clean of blood is boiled and cut up into small pieces. One gram of the coagulated placental tissue is added to one and a half cubic centimetres of haemoglobin-free serum derived from a pregnant woman. The mixture is then received within a dialyzing membrane and placed into twenty cubic centimetres of distilled water. The containers are then incubated for 16 hours. At the end of this time, ten cubic centimetres of the dialysate is tested with two-tenths cubic centimetres of a one per cent aqueous solution of ninhydryn. Ninhydryn gives a blue color in the presence of peptone and amino acids. The appearance of a blue color upon boiling the dialysate with ninhydryn is regarded as postive. Control tests are also run through.

The author has performed three hundred tests and obtained postive reactions in cases one month along up to the end of pregnancy. Five cases were postive four days after absortion, and nine cases were postive one to eight days of the puerperium.

The author is of the opinion that the test may be positive as early as the first day after the fertilization of the ovum. He bases this opinion on the appearance of invertin in the blood serum of animals fifteen minutes after intravenous injection of cane sugar.

H. S. BERNSTEIN,
Editor, Albany Medical Annuals.

### THE DIFFERENTIAL DIAGNOSIS OF SYPHILIS, TUBERCULOSIS, TUMORS AND OSTEO-MYELITIS OF THE LONG BONES.*

Walter M. Brickner, M. D.,

Adjunct Surgeon, Mount Sinai Hospital; Attending Surgeon, Philanthropin Hospital, New York City.

As a plea that some important features of radiographic diagnosis deserve the serious attention of medical—and especially surgical—men generally, I beg to introduce my thesis by repeating here an earlier expression on

"The Interpretation of Radiographs."

"Cystoscopy has become so important a procedure in the diagnosis of diseases of the urinary tract that many surgeons have made themselves expert in its employment and are quite able to make their deductions from it, without the aid of specialists in the art. Radiography is employed much more often and in a much wider range of affections than is cystoscopy, yet how few surgeons have taken the trouble to become expert in the interpretation of x-ray pictures!

"To be sure, all surgeons see so many radiographs that they are quite able to diagnose from them the coarser lesions of the bones and of the thorax. Most of them are quite able to recognize the shadow of a calculus and many are shrewd enough to distinguish from it the shadow of a phlebolith in the pelvis. But in the more delicate interpretation of the x-ray shadings they all too often rely on the dicta of radiographists—many of

*In part, from the surgical services of Dr. Lilienthal, Mt. Sinai Hospital. Some of the x-ray pictures have been courteously furnished by Drs. L. Jaches and I. S. Hirsch, radiographists, respectively, to Mount Sinai and Bellevue Hospitals, New York, and by Dr. Martin Ware.

Read, by invitation, at the Clinical Meeting of the Mount Sinai Hospital, Boston, February 17, 1913.

whom are men of limited scope and few of whom are balancing their work with daily bedside experience.

"To cite a concrete example: A patient is suffering with a pathological fracture of one of the long bones and physical signs fail to show just what the underlying lesion is. It may be, for example, a gumma, a sarcoma, or a carcinoma. The skiagraphs of these three bone diseases may look so much alike that a radiographist not very alert and experienced is apt to make the wrong diagnosis, and on his report a patient may be submitted to a mutilating operation for sarcoma who should have been treated by salvarsan and mercury, or managed conservatively because his carcinoma is metastatic. But bone sarcoma, cancer, gumma, however much they may look alike radiographically, can be distinguished radiographically by expert interpretation of the smaller details of the picture. Each of these lesions has certain characteristic and distinguishing features.

"We often hear men say, 'O, the x-ray is so misleading,' and they are, in truth, often misled, not by the x-ray, but by the faulty interpretation of its unerring traceries. No surgeon has provided himself with his full diagnostic equipment until he learns expertly to interpret these traceries."**

That surgeons, and even many radiographists, so often fail to recognize the skiagraphic features of bone syphilis is not surprising when we find that several x-ray text-books describe them insufficiently and that most surgical works say little or nothing concerning them. In Bevan's translation of Lexer's "General Surgery," published in 1908, the paragraph on diagnosis of syphilis of the bone dismisses the x-ray aspects with the sentence, "Roentgen-ray pictures reveal nothing characteristic" —which statement is quite the reverse of the truth.

The radiographic features of bone syphilis are so characteristic that in most cases the diagnosis can be made from the x-ray plate alone, without, or in the face of, clinical appearances and blood reaction. The exceptions to this rule are probably not more numerous than the exceptions to many other diagnostic signs and reactions.

**Editorial, American Journal of Surgery, November, 1912.

I base this broad statement not only on my own experience, but also on the authority of other observers. Hahn and Deycke-Pascha, (1) Alban Kohler (2) and Martin Ware (3), who were probably the first to formulate the radiographic tracings of bone syphilis, in 1906 and 1907, are likewise emphatic concerning their diagnostic value. I would amplify the deductions made by these writers by suggesting that in those cases in which the diagnosis cannot be made from the radiogram the characteristic features are obscure rather than absent, and to a careful observer the plate will at least sufficiently indicate the possibility of syphilis to suggest a therapeutic or blood test.

Next, of course, to the microscopic study of the tissue removed by incision, radiography supplies the most reliable differentiation between syphilis, osteomyelitis, sarcoma, carcinoma, cyst and tuberculosis of the bones. And because the skiagraphic features that distinguish these have not been generally learned, it is my purpose here to very briefly describe and contrast them so that "he who runs may read:"

SYPHILIS of the bones is essentially a productive inflammation of the periosteum and the bone itself. Two reactions characterize its radiographic picture. The most constant and most distinctive feature is thickening of the periosteum. The second and next most important feature is the thickening of the bony tissue, especially the cortex. Both of these produce black shadows in the radiogram. A third type of process, which may appear, by contrast, as a light area, is gummatous destruction of the bone.

The Periostitis may appear as a localized shadow (fig. 2) or in scattered areas (fig. 1), or it may cover the entire length

1 Hahn—Congress der Deutschen Roentgen Gesellschaft, 1906.

Hahn und Deycke-Pascha—Knockensyphilis in Rontgenbild, Fortschritte auf dem Gebiete der Rontgenstrahlen, Erganzungsband 14. Hamburg, 1907. The reader is especially referred to this excellent monograph and atlas.

2 Kohler—Typische Rontgenogramme von Knochengummen Fortschritte auf dem Gebiete der Rontgenstrahlen, Bd. X, 1906-7, pp. 73-77.

Kohler—In Lehmann's med Atlanten, Bd. VII, Atlas and Grundriss der Rontgendiagnostik, herausgegeben von Franz Groedel, Munchen, 1909.

3 Ware—Annals of Surgery, August, 1907, pp. 199-205; Surgery, Gynecology and Obstetrics, January, 1908, pp. 9-14.

of the diaphysis (fig. 3). It may appear over a circumscribed surface of the bone or, very often, it develops circumferentially (figs. 3 and 4). The shadow may be narrow, fairly parallelling the border of the corticalis (figs. 3 and 4)—"periostitis simplex luetica," or it may be broad and irregular; but in whatever form it appears its outline is distinct (contrast periosteal sarcoma).

Syphilitic periostis begins next to the bone, as a subperiosteal infiltration. In an early stage, therefore, the radiogram may show the periosteal shadow, at this time usually narrow, lifted away from the bone. As the inflammatory process advances the periostitis (and its x-ray shadow) increases, often as the predominating lesion. It may thus acquire considerable density, as in the localized "periostitis gummosa." If a periosteal gumma produces a palpable mass the bulk of this mass being only infiammatory, may cause no shadow on the plate. (Compare periosteal sarcoma.) A periosteal gumma may break down at the site where the inflammatory process is more active, viz., subperiosteally, as in case II, described below. The reaction more often however, takes the form of ossification—also beginning in the deepest layer,—the periosteal shadow then acquiring the density of bone shadow. In the ossifying process periosteum and cortex may fuse into a bony mass, appearing in the picture as a uniformly dense, structureless shadow—ossifying periostitis (fig. 6).

Ostitis. While in some cases of bone syphilis, notably in the early stages, the radiograph shows only the characteristic periostitis, in most there develops, or is seen at the outset, an also characteristic ostitis, marked by thickening of the substantia corticalis or of both the compacta and spongiosa. This process may be localized (fig. 5), or diffuse (fig. 3). The overgrowth of cortical bone may extend only externally (figs. 5 and 6), or internally, encroaching on or obliterating the medulla (fig. 1), or both externally and internally, sometimes as a symmetrical fusiform enlargement. At the site of the ostitis the bone becomes denser (sclerosis), producing a correspondingly denser shadow, in which the bone structure is lost.

The ossifying process may involve the periosteum, fusing bone and bone covering in a solid mass, as above described. In long-standing cases the overgrowth of bone in its axial direction produces the elongation and bowing of the shaft described by Fournier, which is also characteristic of syphilis.

Gummata within the bone (ostitis,-osteomyelitis,-gummosa) when, instead of resolving, they go on to bone destruction, produce light areas in the x-ray picture, but these are surrounded by a dark shadow of reactive bone thickening, which quite distinguishes them from the light areas of bone absorption seen in radiograms of osseous tumors and tuberculosis. The plate* will also reveal thickened bone substance and periostitis. Gummata are usually single in a bone, but sometimes multiple or confluent. They may be very small or involve enough of the bone thickness to cause pathological fracture (fig. 1). Occasionally the gumma surrounds an island of bone, causing it to appear in the radiograph as a sequestrum. But sequestrum formation is unusual in unmixed syphilitic osteomyelitis, which also contrasts it with tuberculous and pyogenic osteomyelitis.

The Osteo-chondritis of hereditary syphilis, which may be the only lesion found in fetuses, and which is sometimes accompanied, in infants, by pseudo-paralysis, also produces characteristic x-ray pictures, as demonstrated by Carl Hochsinger (4). He found in fetuses and infants the following radiographic features: Marked thickening of the epiphyseal end of the diaphysis, the border of which appears very jagged instead of smooth; periostitis over the epiphyseal end of the diaphysis, the shadow varying in density in proportion to the deposit of osteophytes; irregular absorption of bone within the epiphyseal end of the diaphysis; the area of the epiphyseal cartilage much widened and with jagged borders; a shadow of callus formation if there has been epiphyseal separation.

*The terms "light" and "dark" have been used to describe the appearance in x-ray positives. It is best, when possible, to study the negatives.

4 Hochsinger—Die Osteochrondritis epiphysaria im Rontgenbilde. Archiv fur Dermatologie und Syphilis, LVII, 1901, p. 273.

Studien uber die Hereditare Syphilis, Zweiter Teil, pp. 254-298.

Dactylitis (spina ventosa) in hereditary syphilis, essentially an osteo-chronditis, is notoriously difficult to distinguish, by its outward appearance alone, from tuberculous dactylitis. Although the radiogram occasionally shows borderline conditions of doubtful interpretation, it will usually establish the correct diagnosis. Ware has set forth with special clearness the contrasting radiographic features of (hereditary) luetic and tuberculous dactylitis:—

Tuberculosis originates in the epiphysis, syphilis in the epiphyseal end of the diaphysis. In tuberculosis there is little or no peristeal thickening, in syphilis the periostitis is marked. In tuberculosis there is a greater tendency to bone destruction, in syphilis to bone production. In tuberculosis the swelling is largely due to inflammation of the soft parts, in syphilis it is largely due to thickening of the bone. Suppurating sinuses are not uncommon in tuberculous dactylitis, they are in syphilitic dactylitis. Multiple dactylitis is usually not tuberculous, it may be syphilitic or rachitic. "In rachitis, where the pathological changes are also most active at the epiphyseal line, the x-ray generally shows a cup-shaped defect of the epiphyseal parts."

Joint Syphilis is more difficult to diagnosticate radiographically than syphilis of the bone shaft, partly, perhaps, because, being uncommon, one sees too few skiagrams of luetic arthritis to formulate their minuter features, and partly because joint syphilis not infrequently is an inflammation of the soft parts rather than of the osseous tissues. When the latter are clearly invaded, however, they produce the same type of reactions as seen in the x-ray examinations of syphilis of the shaft. In luetic arthritis, then, we note periosteal involvement and bone production. In tuberculous arthritis periostitis is absent or slight, and the bone shows irregular destruction, rather than production or mere rarefaction. Gumma of the articular end of a bone is recognizable in the picture. Destruction of the articular surface by gummous chronditis is, I believe, less easily distinguished. The terminal appearance of a syphilitic arthritis may much resemble, radiographically, that of an arthritis deformans.

In these cases, unless the picture bears the distinctive earmarks of bone syphilis, it is best to arrive at a diagnosis by balancing the radiographic with the clinical findings. Bilaterality of an arthritis or synovitis speaks for syphilis rather than tuberculosis. The anamnesis, the presence or absence of other syphilitic lesions and the reactions to the Wassermann tests cannot here be ignored.

Osteo-porosis, sclerosis, caries are the end products of syphilitic, as of all other infections of the bones. When they are irregularly distributed in an advanced lesion, it may be very difficult to distinguish, radiographically, luetic osteomyelitis from

Chronic Osteomyelitis of Pyogenic Origin. In these conditions, as in syphilis, the x-ray may show well-marked periostitis, but, as contrasted with syphilis, this may be slight or altogether absent. As in syphilis, there is also seen in many cases extensive areas of osteosclerosis and osteoporosis. The sclerosis may be quite dense, but usually is not as uniformly so

Figure 1.—Case I. Gumma of Humerus with Pathological Fracture, Mistaken for Sarcoma.

Note marked periosteal thickening, and contrast with picture of periosteal sarcoma, Fig. 11. Note thickening of bone tissue encroaching on medulla; rarefied area surrounded by dense zone, not by thinned-out cortex; absence of expansion. Contrast endosteal sarcomata, Figs. 9 and 10.

as in syphilis, and may indeed present a "mottled" appearance. The involucrum, often seen in chronic pyogenic osteomyelitis, does not occur as such in syphilis and, as compared with the new bone produced in the latter, it usually is thinner, more "porous"

—at least in places—and shows in part or throughout some details of bone structure (Fig. 18); its borders, also, are thin and

Figure 2.—Case II. Gummous Periostitis of Femur, Mistaken for Sarcoma.

Note sharp periosteal shadows, increased density and thickening of bone substance, and absence of shadow of the palpable mass.

irregular. Extensive destruction or absence of bone cortex is commonly seen in pyogenic osteomyelitis, presenting an image that we do not see in lues. Sequestra, large and small, are common in pyogenic osteomyelitis, unusual and small in syphilis. Large, freely discharging bone sinuses, common in pyogenic osteomyelitis, are very uncommon in syphilis and when a sinus does occur here it is apt to be small and to discharge scantily. Central abscesses, marked by light areas in the picture of chronic osteomyelitis, resemble very closely similar areas

Figure 3.—Case III. Syphilitic Osteo-Periostitis of Humerus with Gummata of Soft Parts, Mistaken for Pyogenic Osteomyelitis.

Note smooth, sharp outline of circumferential periostitis along the shaft, and irregular overgrowth of bone substance encroaching on medulla.

produced by small gummata. A sequestrum may be seen in either, but more often in the former. While the zone surrounding a gumma is apt to appear denser than that about a

Figure 4.—Case IV.—Gumma of Head of Humerus. Early lesion. Note fine lines of periostitis (marked by single arrows), and beginning rarefaction and necrosis in the bone (marked by double arrows.)

Figure 5.—Old Localized Syphilitic Osteo-Periostitis (Gumma) of Tibia.

Note marked bone growth, sclerosis and areas of porosis. Periosteum ossified with cortex.

bone abscess, it is best to make the differential diagnosis by other radiagraphic stigmata. The picture of chronic osteomyelitis ought not to be confused with that of a bone tumor, the features of which are described below.

Figure 6.—Old Syphilitic Osteo-Periostitis of Tibia.

Note bone growth, sclerosis, and porotic area below tibial tubercle.

Figure 7.—Syphilis of Lower End of Radius.

Periosteal shadow somewhat resembles that of periosteal sarcoma, but the condition is differentiated by the marked enlargement and increased density of the bone.

TUBERCULOSIS in its differential radiographic features has been referred to. It occurs in the diaphysis very rarely and then only as an extension from the epiphysis. Here it causes rarefaction, small areas of necrosis along the articular surface

(if extending from the synovia) or within the bone, or extensive destruction of the epiphysis or cartilages. There is little or no periosteal reaction.* Sequestra are usually cortical. In rather advanced cases there is often an extensive bone atrophy (absorption of lime salts) which appears clearly in the plate, and involves neighboring bones. While this extensive bone atrophy

Figure 10.—Central Sarcoma of Radius with almost complete disappearance of bony structure.

Figure 11.—Periosteal Sarcoma of Tibia.

Note blotchy periosteal shadow thinning out in its periphery, also faint shadow of the tumor outline (compare Fig. 2), and absence of change in the bone.

is not often seen in pyogenic osteomyelitis, it is not at all pathognomonic of tuberculosis; e. g., in Fig. 13, from a carcinoma of the ulna, the humerus is seen much rarefied. The picture of bone tuberculosis may resemble that of neoplasm, if there is a fairly outlined area of rarefaction (Fig. 17). Physical signs and symptoms will help to differentiate in such a radiographically doubtful case.

*Kohler states that massive ossifying periostitis is seen in long-healed bone tuberculosis.

SARCOMA of the bones may be divided, for our purposes, into its two main types—central or endosteal, and periosteal.

ENDOSTEAL SARCOMA presents a characteristic picture: marked rarefaction in the tumor area, expansion of, and shell-

Figure 12.—Carcinoma of Head of Humerus, first form.

Note resemblance to central sarcoma, but absence of expansion.

Figure 13.—Carcinoma of Ulna, second form, Case V.

Note eaten-out, porous, "spongy" appearance of the ulna, absence of bone production and of periosteal reaction. Note also the atrophy of the humerus.

like thinning out of the cortex. There is no periosteal shadow while the tumor is within the bone (Fig. 9) and there may be none even if it breaks through the bone (Fig. 10). At the site of the tumor all cortical structure may be so destroyed that none appears in the radiogram.

As contrasted with gumma the cortex about a sarcoma is thinned and expanded, not thickened, there is no dense zone about the light area, and no periosteal deposit.

Periosteal sarcoma presents a periosteal shadow for which the picture of syphilis is often mistaken by the unwary, as in Cases I and II. The periosteal image in syphilis is, however, sharply outlined and may be quite dense, while that in periosteal sarcoma is irregular and thins out in its periphery where it is lost in the surrounding tumor area, which also casts a shadow, however faint (Fig. 11. Contrast periosteal gumma, Fig. 2, with no shadow of the mass). The shadow in sarcoma is often blotchy, wavy or fringe-like and, instead of hugging the bone, as in syphilis, it may appear to radiate from it like miniature swirls in a sandstorm. One may, however, sometimes see a fringe-like periosteal shadow in syphilis, as in Fig. 7, but it

Figure 14.—Cyst of Humerus, with Pathological Fracture.

Note rarefaction in cystic area, thinning and expansion of cortex the same as in central sarcoma. Contrast sharp outline of cyst, and clear shadow of trabeculæ, not seen in sarcoma.

can be distinguished from that of periosteal sarcoma by the accompanying changes in the appearance of the bone itself. In periosteal sarcoma the shadow of the bone may appear normal, or merely darkened by the overlying tumor. If the growth invades the corticalis, its shadow is correspondingly intensified and widened. Chrondrification or ossification in a periosteal sarcoma appears in the picture according to its extent.

Figure 15.—Cyst of Lower End of Tibia.

Note rarefaction, thinning out of cortex, sharp outlines, and trabeculæ.

Myeloma presents the same x-ray appearance as endosteal sarcoma, with which it is often grouped, clinically and pathologically. It has a well-known tendency to multiplicity and a predilection for the ends of the long bones, the ribs and the cranial diploe.

Carcinoma, in my exper[illegible]nce, appears in the bones, radiographically at least, in two forms. In one the picture is much like that of a central sarcoma, viz., rarefied tumor area and thinning of the surrounding bone, with this difference, however, that in carcinoma there is absence of the expansion of the bone shell so conspicuous in sarcoma (Fig. 12).

In the second form the radiogram shows the bone irregularly eaten away, superficially as well as in the depth, and what bone structure is left in the tumor area appears spongy, porous, eroded (Fig. 13). In carcinoma one never sees reactive bone production.

Bone Cysts, of various types histologically, present an x-ray picture much like that of endosteal sarcoma: translucency; thinning and expansion of the cortex. As compared with sarcoma, however, the bony wall of the cyst is sharply outlined (a

Figure 16.—Multiple Enchondromata.

Note rarefied areas, thinning and expansion of cortex, trabeculation of the tumors, their extension from epiphyseal lines, and the multiplicity of the growth.

diagnostic feature which, of course, is lost if the picture is blurred by a movement of the patient during exposure); and the light area is often traversed by the shadows of trabeculæ (Figs. 14 and 15). Trabeculæ are sometimes seen in central sarcomata, but they do not appear as sharp or as complete as in cysts. The wall of the bone cyst is often the seat of a pathological fracture, but the radiogram never shows it extensively absorbed or broken through as in sarcoma (Fig. 10).

Enchondroma provides an x-ray picture like that of central sarcoma and that of cyst: translucent area, thinning and expansion of the cortex. The light area is apt to appear distinctly lobulated (trabeculated); its border is fairly sharp; it extends from the line of the epiphyseal cartilage; and in many cases it will be found in two or more bones (Fig. 16).

Figure 17.—Tuberculosis of Head of Humerus.

Note the cyst-like area of bone absorption, surrounded by an area of bone atrophy. Note also the atrophy in the glenoid, acromion and coracoid. The patient had pus in the tissues about the joint and tuberculous cervical lymph nodes.

### Illustrative Cases.

Case I.—J. G., a chauffeur, about 30 years old, suffered a fracture of the midshaft of his right humerus while shifting gears in driving an automobile. There were distinct crepitus and slight thickening at the site of the pathological fracture, and a clinical diagnosis of sarcoma was made by several surgeons.

They were confirmed in their opinion by the x-ray picture (Fig. 1), on which an experienced radiographist also reported unequivocally "sarcoma;" and the patient was prepared for operation. After scrutinizing the picture, and before examining the patient, I insisted that the lesion was not a sarcoma, but a gumma.

The radiogram presents the characteristic periostitis of bone syphilis and the also characteristic bone production and thickening of the bone substance. As opposed to periosteal sar-

Figure 18.—Chronic Pyogenic Osteomyelitis of Ulna.

Note the extensive involucrum, showing considerable bone structure, formed in an effort to reproduce the entire shaft. The diseased shaft shows necrosis, absorption, fracture, but no bone production within itself and no periostitis. Compare Figure 8.

coma: the periosteal shadow is heavy, distinctly outlined, hugs the bone and does not fade off in the periphery; there is no shadow of the large tumor mass that so extensive a periosteal neoplasm would have developed; there is a translucent area within the bone and a pathological fracture through it, neither of which is found in periosteal sarcoma. As opposed to central sarcoma:

the translucent area is surrounded by a much thickened, not a thinned corticalis; below the light area the medulla appears almost obliterated by the growth of new bone; there is no bone expansion; there is a periosteal reaction not seen in endosteal sarcoma.

A clear history of syphilis was then elicited from the patient, and his blood yielded a positive Wassermann reaction. After two injections of salvarsan the bone fragments united in four weeks, and, specific treatment being continued, the patient was soon restored to health and activity with arms sound and whole.

Case II.—G. M., male, aged 50, was suffering with a painful, slow growing, fist-sized, fusiform, smooth, hard, not tender mass, firmly attached to the upper third of the femur, anteriorly. There was no history of syphilis; Wassermann reaction negative. A clinically justifiable diagnosis of osteosarcoma was made, and an experienced radiographist reported his finding (Fig 2) "periosteal sarcoma." Here, again, I insisted that the radiogram was typically that of a luetic process, in spite of the negative blood finding, and that the large mass was therefore a gumma. The picture shows characteristic periosteal reaction and dense shadow of bone production. A periosteal neoplasm causing a swelling of such size would have cast some shadow on the plate, and the periosteal outline would have presented a different appearance.

After intravenous salvarsan injection the Wassermann reaction was again negative. The patient continued to suffer considerable pain and to exhibit a subfebrile temperature course, and he developed a leucocytosis of 18,000—for all of which reasons I thought there was some breaking down or suppuration and decided to open through the mass. The operation revealed periosteum of half-inch thickness and breaking-down of the gumma subperiosteally. The bone beneath was trephined over an area of two inches and the medulla was found normal. A section from the periosteum, and the grumous subperiosteal material was reported "syphilitic inflammation." The drainage relieved the man's pain, and under continued specific treatment he has been making a good recovery.

CASE III.—A boy of 10 suffered gradually increasing pain in the left arm, which was somewhat swollen and slightly tender, and showed at its upper end, externally and internally respectively, a rounded, raised, reddened area discharging thin pus through a sinus. Two surgeons had diagnosed osteomyelitis and advised operation. The two discharging swellings appeared to me to be gummata and suggested to me that the underlying bone lesion was syphilitic. The x-ray picture (Fig. 3), which a very competent radiographist reported "osteomyelitis," made me sure of my clinical diagnosis, in spite of a negative Wassermann reaction and absence of syphilitic history. The picture shows beautifully the sharply outlined tracings of periosteum circumferentially thickened along the diaphysis. The somewhat scalloped, irregular border between the medulla and the bone proper, which had been interpreted as indicating myelitis, is due to irregular encroachment on the medulla by the process in the compact and spongy portions. Under specific treatment the boy recovered without other operation than incision and drainage of the gummata.

CASE IV.—J. J., aged 32, was referred to me several years ago because of severe pain and disability in his left shoulder. I was one of several surgeons who incorrectly diagnosed his ailment, which was variously considered periarthritis, subacromial bursitis, tuberculosis and sarcoma. Several months after I saw the patient a small section removed from the head of the humerus was reported "chronic inflammation," and eventually the disease was proved to be syphilitic. If to-day the radiograph (Fig. 4) taken when I first saw this man, were submitted to me, I should recognize in the fine tracings of periostitis about the neck of the humerus the tell-tales of syphilis, and in the beginning rarefaction and erosion in the region of the tuberosities, associated with the periostitis, the imprint of a gumma.

In CASE V also I was one of several surgeons who failed to make a correct clinical diagnosis. The patient was an elderly, emaciated woman admitted to the second surgical service of

Mount Sinai Hospital with gradually developing pain in the right forearm, the ulna aspect of the upper end of which was swollen, edematous and tender; the elbow joint was normal. The condition was variously diagnosed chronic osteomyelitis, tuberculosis, syphilis and sarcoma of the ulna. The radiogram (Fig. 13) was also variously interpreted. A section of the bone and soft parts which I curetted from the forearm through a small incision was reported carcinoma. Subsequent observations indicated that the woman probably had a primary growth in the stomach.

Case VI.—D. S., painter, aged 40, presented himself seven years ago in my then out-patient surgical department of Mount Sinai Hospital with a fracture of the mid-shaft of the right humerus, acquired in falling from a scaffold. No radiograph was made, for reduction was easily maintained and the patient made a very satisfactory recovery. A year later he returned to me with the report that a few hours before he had been jostled in a car and felt the right arm break again. I found it broken, indeed, and made a diagnosis of sarcoma on the history. The x-ray plate showed an extensive central sarcoma involving the greater part of the humeral diaphysis, as indicated in Fig. 9.

In the hospital, February, 1907, Dr. Lilienthal removed the entire growth, leaving only the upper and lower ends of the bone. After ultimate healing, a metal brace was provided for the arm, which allowed the patient to make good use of his extremity. After six years without any recurrence of the growth (round-cell sarcoma), the patient was re-admitted to the hospital in November, 1912, and I introduced into his arm a long graft taken from his left tibia, bridging the large space between the upper and lower fragments of humerus. Unfortunately, the wound became infected (perhaps because a large number of visitors crowded about the operating table) and the graft had to be removed later.

This case is referred to, not because it represents a mistake in diagnosis, but because it illustrates: first, a rare condition—sarcoma developing at the site of a fracture; and, second, the excellent result of a conservative operation (resection) for bone sarcoma, as compared with amputation. It is to be regretted that it does not also illustrate a good result in a homologous grafting to replace a large bone defect.

30 West 92nd Street.

---

### THE TREATMENT OF AMEBIC DYSENTERY WITH SUBCUTANEOUS INJECTIONS OF EMETINE HYDROCHLORID

(Report of Six Cases)

Randolph Lyons, M. D., New Orleans
*Instructor in Clinical Medicine, Tulane University*

A new treatment of amebic dysentery is of particular importance to physicians in the Gulf States because of the frequency with which the disease is met in practice. It was therefore with much interest that I read the reports of the treatment of amebic dysentery with soluble salts of emetine by Dr. Leonard Rogers of India. My clinical experience with ipecac in amebic disease, while limited to thirty cases, led me to look on this drug as practically specific when administered in sufficiently large doses (60 to 100 grains daily).

In 1911 I made some experiments,[1] at the suggestion of Dr. George Dock, to test the specificity of ipecac on amebas *in vitro.* These experiments failed to demonstrate any specific action, probably because the ipecaca used, as I later discovered, as much below the pharmacopeial standard in total alkaloids (emetine, etc). Still another cause of failure was the employment of watery suspensions of ipecac, in which the drug is but slightly soluble. About a month previously, Vedder,[2] working independently in the Philippines, reported his experiments with ipecac in which he showed that emetine was in all probability the active principle of ipecac as regards its destructive action on amebas.

1. Lyons, Randolph: Observations on the Effect of Ipecac, Phenol and Salicylic Acid on Amebas in Vitro, Read Before the Am. Soc. Trop. Med., 1911.

2. Vedder, E. B.: A preliminary Account of Some Experiments Undertaken to Test the Efficiency of the Ipecac Treatment of Dysentery, Bull. Manila Med. Soc. March, 1911.

### Rogers' Emetine Treatment

Using Vedder's experimental findings as a basis, Rogers developed to emetine treatment. His first article[3] appeared in June, 1912. In this he reported three cases treated hypodermatically with emetine hydrochlorid with striking success. In August of the same year[4] he published his further experience of the specific, curative action in amebic diseases of the hypodermic injections of soluble emetine salts. Twelve cases were described with two deaths. At necropsy gangrene of the bowel was found in one multiple liver abscesses in the other. Of the remaining cases (cures), two were complicated with heptatis, two with single liver abscess and one with splenic abscess. The next publication appeared in October.[5] In this Rogers compares twenty-four cases of amebic dysentery treated with soluble salts of emetine, with thirty consecutive cases in which ipecacuanha was used. The following tabulation shows the result:

| | Ipecac | Emetine |
|---|---|---|
| Died from dysentery within 3 days of admission | 4 patients | 2 patients |
| Died from dysentery over 3 days after admission | 7 patients | 0 patients |
| Died from other diseases after cure of dysentery | 0 patients | 2 patients |
| Removed from hospital in very bad condition..... | 2 patients | 0 patients |
| Left hospital unchanged or not cured .......... | 4 patients | 0 patients |
| Cured .......................................... | 13 patients | 20 patients |
| Average days in hospital of cured patients ..... | 16.4 | 7.2 |
| Average days before stools became normal .... | 11.4 | 2.35 |
| Grains of ipecac or emetine to that time ........ | 406 | 2. |

Rogers' last article[6] appeared in December, 1912. Here the new treatment is reviewed and discussed. Still more re-

3. Rogers Leonard: The Rapid Cure of Amebic Dysentery and Hepatatis by Hypodermic Injections of Soluble Salts of Emetine, Brit. Med. Jour., June 22, 1912, p. 1424.

4. Rogers, Leonard: Further Experience of the Specific Curative Action in Amebic Disease of Hypodermic Injections of Soluble Salts of Emetine, Brit. Med. Jour., Aug. 24, 1912, p, 405.

5. Rogers, Leonard: Amebic Colitis in India: Prevalence, Diagnosis and Emetine Cure, Lacent, London, Oct. 19, 1912, p, 160.

6. Rogers, Leonard: The Rapid and Radical Cure of Amebic Dysentery and Hepatitis by the Hypodermic Injections of Soluble aSlts of Emetine, Therap. Gaz., Dec. 15, 1912, p, 837.

cently Allen[7] reported two cases of amebic dysentery treated with emetine. Both patients were apparently cured. The second case required 12.3 grains of emetine hydrochlorid. No mention is made in regard to the subsequent history of the patients.

After careful perusal of Rogers' papers, one cannot help being impressed by the rapid results obtained by the new testament. Certain questions, however, arise: First, what are the shortcomings of the older ipecac treatments? Second, what are the supposed advantages of Rogers' method of treatment? Lastly, does it cure?

### Shortcomings of Older Methods

No matter how enthusiastic one may be in regard to the treatment of dysentery with ipecacuanha, it must be admitted that the modes of administration are crude and cumbersome. Because of its emetic action, to give it in liquid form is practically out of the question. To overcome this objection, the powdered drug is put up in salol or keratin-coated pills in order that they may pass through the stomach undissolved. Of the two coatings salol (phenyl salicylate) is to be preferred because of its bactericidal and amebicidal properties. Each salol-coated pill contains 5 grains of ipecac and the dose of ipecac is from 60 to 100 grains (adult) daily for at least a week, that is, from twelve to twenty bulky pills each night. This in itself is a formidable undertaking and may sometimes, in nervous patients, produce emesis. If the coating of a pill is defective or too thin there will be vomiting; on the other hand, if the coating is old or very hard many pills may pass through the intestinal tract intact. This applies especially to machine-made salol-coated pills put on the market by pharmaceutical houses.

Recently Beck[8] advised the duodenal medication of ipecac by means of an Einhorn duodenal tube. Such a procedure may insure the passage of the drug into the duodenum but could scarcely be called an improvement in method of administration.

7. Allen, William: The Emetine Treatment of Amebic Dysentery, The Journal A. M. A., March 1, 1913, p. 664.

8. Beck, H. G.: Duodenal Medication of Ipecac in the Treatment of Amebic Dysentery, The Journal A. M. A., Dec. 14, 1912, p. 2110.

Lastly, unless the ipecac has been previously assayed it may be found below the U. S. P. standard. This unreliability of the drug is a serious handicap in its successful usage.

Before discussing the emetine treatment it may be well to describe briefly the cases observed and their treatment. I had the preparations made up by a local chemist from the alkaloid emetine. This product caused, at times, considerable local irritation and infiltration owing to a slight excess of acid, and absorption was doubtless on these occasions much delayed. In fairness to the drug, allowances for this fact should be made in two of the cases.

In order to give the treatment a thorough test no other medication was employed and no colonic irrigations. Cases were not selected and all patients were kept in bed on liquid or soft diet while taking the injections.

### Brief Report of Cases

Case 1.—M. F., colored man, aged 27, carpenter, was admitted to Charity Hospital August 15, 1912. Patient's first attack began about January 7, 1912, and has persisted up to date of admission with the exception of a period of improvement of three weeks in May. The stools varied in number between ten and twenty in twenty-four hours, often accompanied with tenesmus and containing menus, pus and blood.

Treatment and Course.—Patient given ipecac in 5-grain salol-coated pills, beginning with 75 grains first night; dose gradually reduced to 60 grains. The length of treatment was seven days; total amount of ipecac 445 grains minus four pills passed in stools equal to 425 grains. Bowels were apparently controlled but some abdominal tenderness and discomfort remained and patient went home August 27 against my advice.

October 4 patient was readmitted to hospital having first been examined at my office. He stated that diarrhea began again six days previously and admitted that he had never been free from slight discomfort in abdomen since leaving hospital. He had passed 17 stools in past twenty-four hours. Examinations of stool showed ameba, pus, mucus, blood and trichomonads.

Physical Examination.—Temperature normal; pulse, 76; fairly well nourished; mucous membranes slightly anemic; abdomen, flat; tenderness in left iliac region; general moderate adenopathy.

Further Treatment and Course.—Emetine hydrochlorid 2-3 grain subcutaneously in arm at 9:30 a. m.; following this he had ten stools.

October 5: This forenoon patient had soft movement; no tenesmus.

October 6: Seat of injection shows slight infiltration and is tender. Only three stools; no blood or pus noticeable.

October 7: One soft stool this forenoon; contains no ameba or blood. Emetine hydrochlorid 5/8 grain injected in other arm. Seat of first injection still a little sore.

October 8: Well-formed stool. Patient up and on full diet. He states his abdomen feels entirely comfortable for first time in six months

October 9: Emetine hydrochlorid 1/2 grain was injected to-day as a safeguard against recurrence.

October 12: Bowels normal for past four days. Patient discharged but urged to report at office.

October 18: (Office) Patient says he has been feeling splendid. Bowels perfectly normal.

February 6, 1913: (Office) Patient fine. Has gained 15 pounds. Bowels normal.

In this case ipecac failed in spite of a course of treatment with moderately large doses. Yet the patient reacted in a very striking manner to two doses of emetine subcutaneously. He has remained well for nearly four months following. Total amount of emetine administered was 1.8 grains, equivalent to about 170 grains of ipecac.

Case 2.—W. L., white man, aged 47, cook, home, Bogalousa, La., was admitted October 9, 1912. He had been on a spree fifteen days previously and following this his bowels became loose. Stools numbered about fifteen per day and contained blood and "slime." Tenderness also was present. He lost considerable weight and felt very weak. He developed a cough during the past two weeks and expectorated freely. He stated he had fever at onset.

Physical Examination.—Temperature, 98; pulse, 70, somewhat weak; low pressure. Lungs, a few sonorous rales heard at right apex anteriorly and posteriorly, otherwise negative. Heart, negative. Abdomen, slightly distended and very tender especially over right iliac region; some rigidity over this area. Patient appears anxious and complains of abdominal pains. Sweat on forehead. He appears a poor subject for a new treatment, as peritonitis seemed more than likely.

Course and Treatment.—October 10: Passed twenty-five bloody stools in past twenty-four hours. Temperature in evening, 100.8.

October 11: Emetine hydrochlorid 2-3 grain. Number of stools slightly less. Sign of peritonitis more evident. Abdomen more tender and rigid; hiccough present; clammy sweat on forehead; pulse 100, small and weak; sputum, negative for Bacillus tuberculosis.

October 12: Emetine hydrochlorid 5/8 grain. Examination of stool, foul; blood, pus, little mucus. No amebas found; necrotic material present.

October 15: Marked bulging of epigastrium; great tenderness; bowels move about every hour but no amebas can be found. Stools have foul odor, and pus, blood and necrotic material as chief ingredients. Patient suffering a good deal. Opiates required.

October 18: An attempt was made October 16 to administer ipecac pills by mouth but patient was unable to swallow them. Patient failed rapidly in past three days and died to-day at 10 a. m.

Partial necropsy permitted. Abdomen only was opened. Necropsy diagnosis: "Amebic dysentery, gangrene of the large intestine, perforation and peritonitis." Practically all of the large intestine, except a few inches of the descending colon, was gangrenous. There was walled-off fecal masses from perforations in the epigastrium, right iliac region and in the rectum.

The necropsy clearly demonstrated the utter hopelessness of any therapeutic measure in a fulminating case of this type. The case, however, illustrated that in very severe types of dysentery a subcutaneous or intravenous method of treatment is the only one offering any possibility of cure. It is unfortunate that the colonic contents and walls were not examined at the necropsy for amebas. It might have been shown that the amebas had been destroyed by the emetine injections, and the gangrenous process was due entirely to secondary invaders, especially in view of the fact that the last three examinations of his stools revealed no amebas.

Rogers has reported a case of very similar history in which no necropsy, scraping of the ulcers and stained sections through the walls of some of the more recent ulcers failed to show a single parasite.

Case 3. E. Y., colored man, aged 25, waiter, was admitted November 25, 1912 Patient taken sick about eight months ago at Biloxi, Miss., with loose bowels and straining. Stools contained blood and "slime." He also suffered from a constant gnawing sensation in abdomen. Bowels have progressively become worse. Number of actions between ten and eighteen in twenty-four hours. Says he feels weak and has lost weight.

Physical Examination.—Early well nourished; mucous membranes pale. Thorax, negative; abdomen, flat, tender in epigastrium and iliac regions. Temperature normal; pulse 70, low pressure. Examination of stool: many motile amebas, blood, mucus and pus; few trichomonads.

Treatment and Course.—November 6: Fifteen stools in past twenty-four hours. Emetine hydrochlorid ⅝ grain, subcutaneously (fresh solution).

November 7: Site of injection sore. Seven stools in past twenty-four hours. Emetine hydrochlorid ⅝ grain by needle.

November 8: Has had five stools since last injection; character of stool changed, now yellowish; contains but small amount of mucus, no blood. Emetine hydrochlorid ¾ grain by needle.

November 9: Tenderness in abdomen nearly gone. Five stools. Examination revealed, after long search, one sluggishly motile ameba, a little mucus, no blood, numerous trichomonads.

November 12: No more injections given because patient complained so much of pain in previously injected muscles. Examination of the emetine hydrochlorid solution showed it to be distinctly acid to litmus, instead of neutral.

November 13: Sites of injection still painful and infiltrated. Four or five stools per day. Examination of stool revealed a few sluggishly motile amebas, encysted amebas, little blood and numerous trichomonads. Because of the pain caused by the new solution of emetine, I decided to give the patient ipecac pills until a fresh solution could be prepared. He was given twelve (60 grains) salol-coated ipecac pills. A few hours later he had a severe vomiting attack.

November 15: Patient refused pills on following night. He was persuaded to try them again but after taking eight pills (40 grains) again vomited and refused to try them any more, stating that he preferred the pain of the injections.

November 16: Emetine hydrochlorid (new preparation) ⅝ grain by needle.

November 17: Seven stools since injection. No pain, no blood.

November 18: One stool. Emetine hydrochlorid ⅝ grain by needle.

November 19: One formed stool. Patient says he feels all right and wants to go home. Discharged after promise to report to office.

November 30: (Office) Has gained 2½ pounds. Says bowels act regularly once a day. Eats anything he wants.

January 21, 1913: (Office) Feels fine; has gained 11 pounds. Bowels perfectly normal.

The number of injections required in this case was probably due to the fact that the dosage was small and absorption very slow owing to the irritation produced by the activity of the first preparation. Infiltration about the site of the injection could be felt as long as ten days.

The fact that the patient was highly nervous may account for the vomiting which followed the administration of ipecac pills. At all events the case illustrates the difficulties sometimes met in giving ipecac by mouth.

Case 4.—J. C., colored man, aged 33, laborer, home, New Orleans, was admitted November 18, 1912. Present illness began in May 1912, with diarrhea. Bowels moved at first five or six times in twenty-four hours; were slimy and accompanied with straining. Some abdominal pain between actions. His bowels gradually became worse. Of late they act as often as fifteen to twenty-five times in twenty-four hours. The stools contain blood and mucus. Patient has constant but slight abdominal pain. Has lost 25 pounds.

Physical Examination.—Rather poorly nourished, slightly anemic. Temperature, normal; thorax, negative; abdomen, upper half, tender; no distention. Liver and spleen not palpable. Examination of stool shows mucus, blood, pus and motile amebas. No trichomonads.

Treatment and Course.—November 20: Patient's bowels moved thirteen times in past twenty-four horus. Emetine hydrochlorid ½ grain by needle in forenoon.

November 21: Six stools since injection. Abdominal pain absent for first time in many months. Emetine hydrochlorid ⅝ gain.

November 22: No stool since last injection, feels much better. Both arms tender from injections.

December 2: Bowels have been normal since November 23 with tendency to constipation. Requires purgatives.

December 12: (Office) Has gained 5 pounds and reports bowels are perfectly normal. Feels fine.

March 4, 1913: (Office) Bowels are normal. Feels perfectly well.

The effect of the injections in this case was very striking. Twenty-four hours after the first injection all abdominal discomfort disappeared and after the second, the stools became normal and remained so. The total amount of emetine administered was 1.1 grains.

Case 5.—S.S., colored man, aged 40, laborer, home, Adeline, La., was admitted November 19, 1912. About the middle of June patient's bowels became loose; fourteen and fifteen actions in twenty-four hours containing bloody mucus and at times accompanied by tenesmus and abdominal pain. Patient has lost about ten pounds.

Physical Examination.—Fairly well nourished. Temperature normal. Chest negative. Abdomen, negative except for tenderness in lower half. Subluxation of right hip. Examination of stool; mucus, blood and pus; few motile amebas; many trichomonads.

November 26: Patient had nine actions in past twenty-four hours. Emetine hydrochlorid ½ grain by needle.

November 27: Five stools since injection and seat of injection tender. Emetine hydrochlorid ⅝ grain by needle.

November 28: Discomfort in abdomen better. Three stools. Both arms tender. Emetine hydrochlorid ¾ grain.

(To be Continued.)

## Figures and Tables

**Figure 1. f1:**
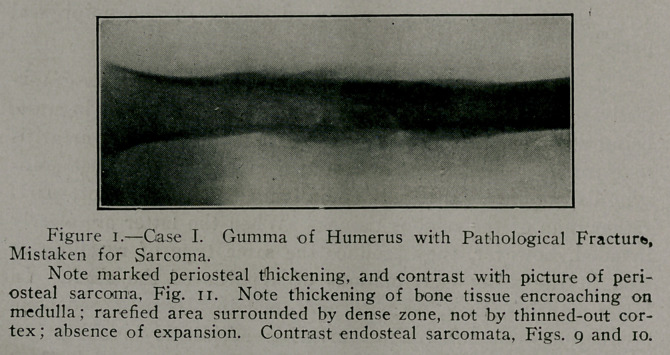


**Figure 2. f2:**
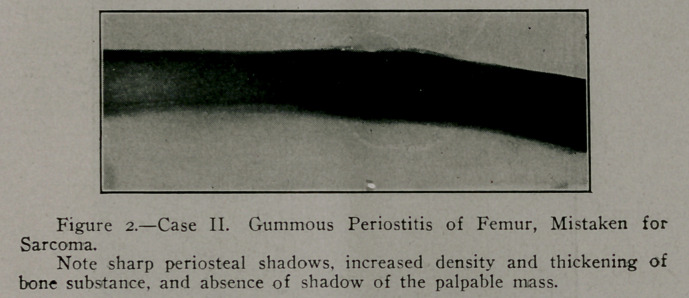


**Figure 3. f3:**
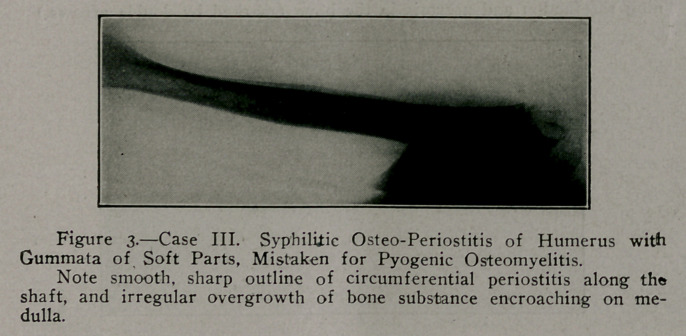


**Figure 4. f4:**
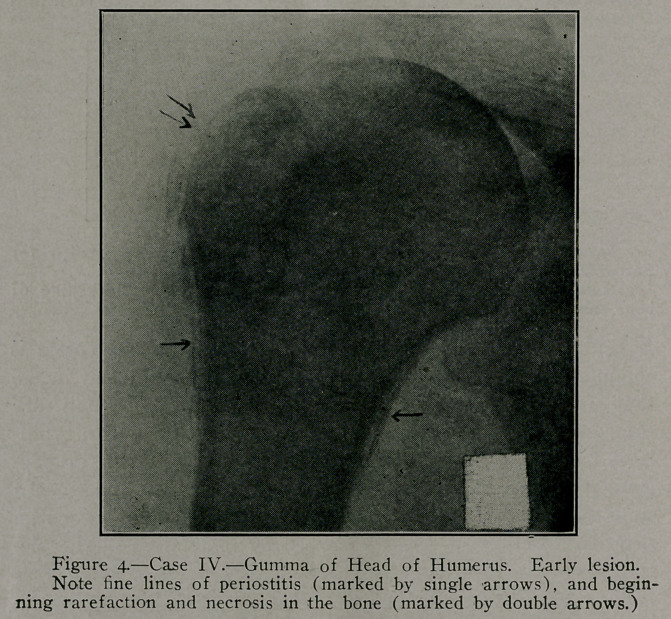


**Figure 5. f5:**
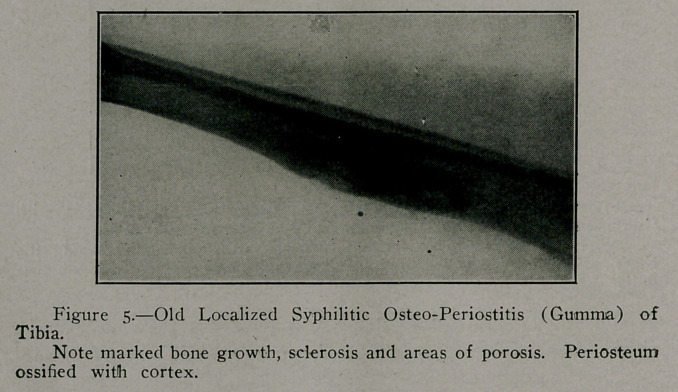


**Figure 6. f6:**
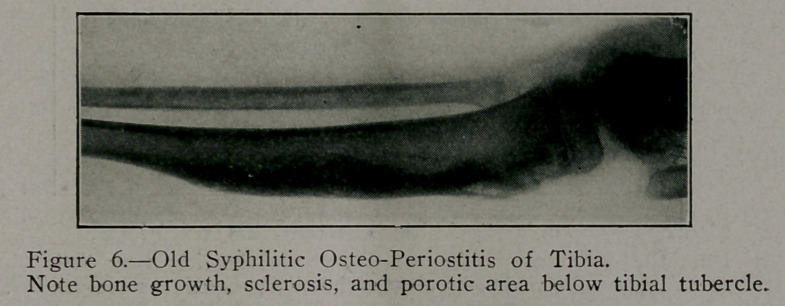


**Figure 7. f7:**
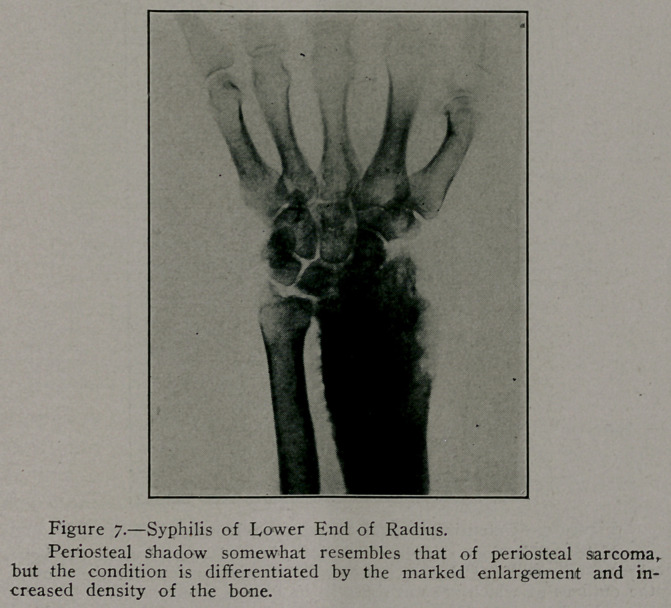


**Figure 8. f8:**
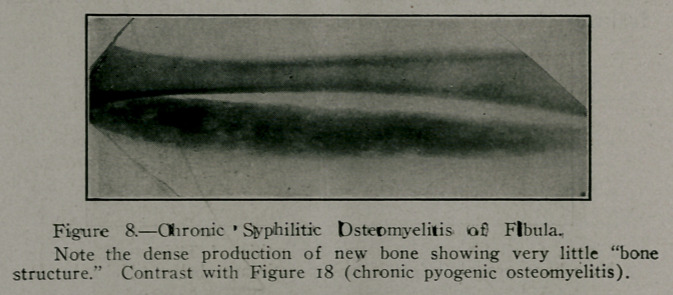


**Figure 9. f9:**
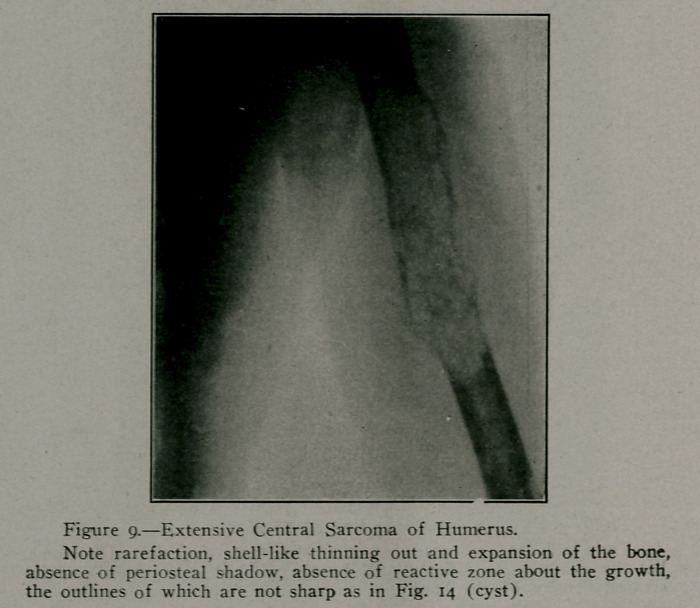


**Figure 10. f10:**
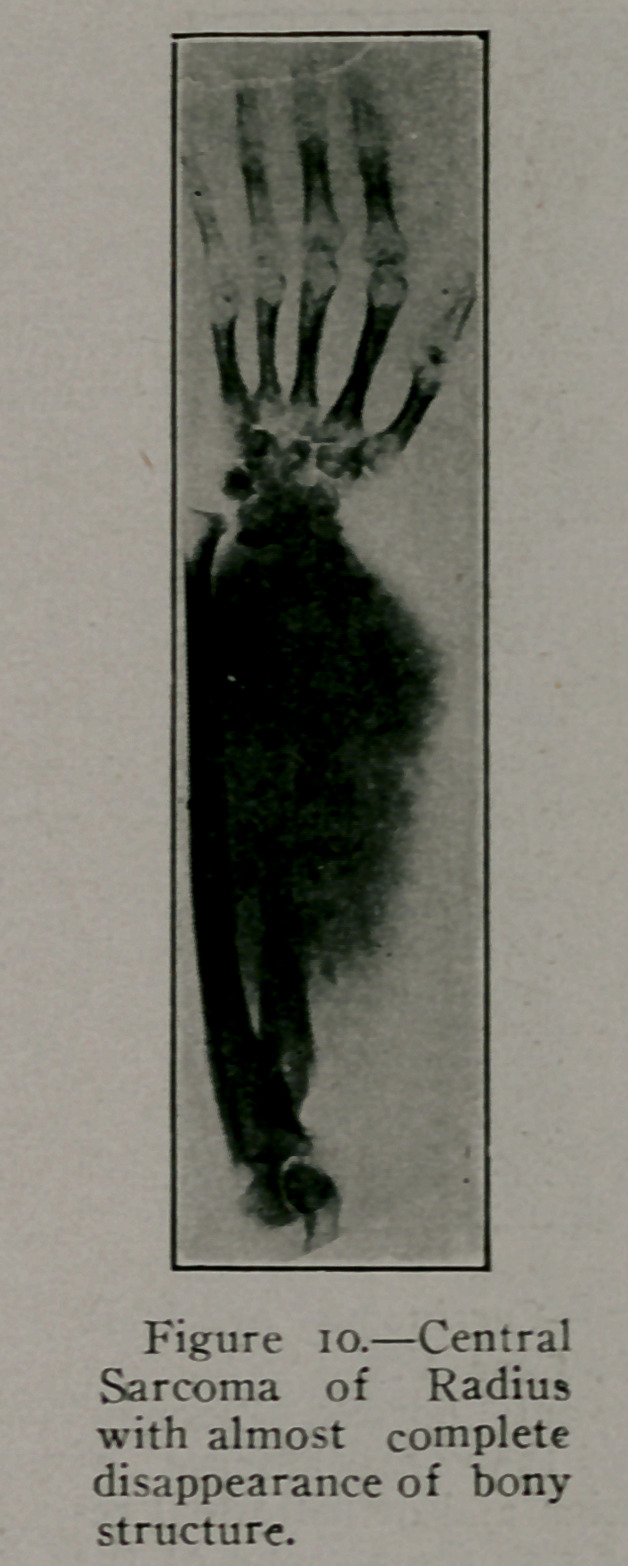


**Figure 11. f11:**
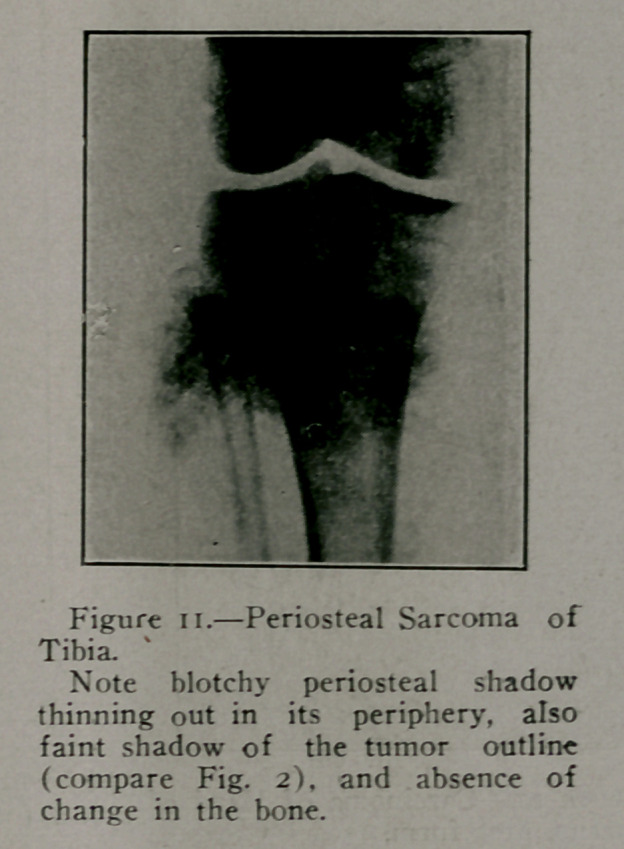


**Figure 12. f12:**
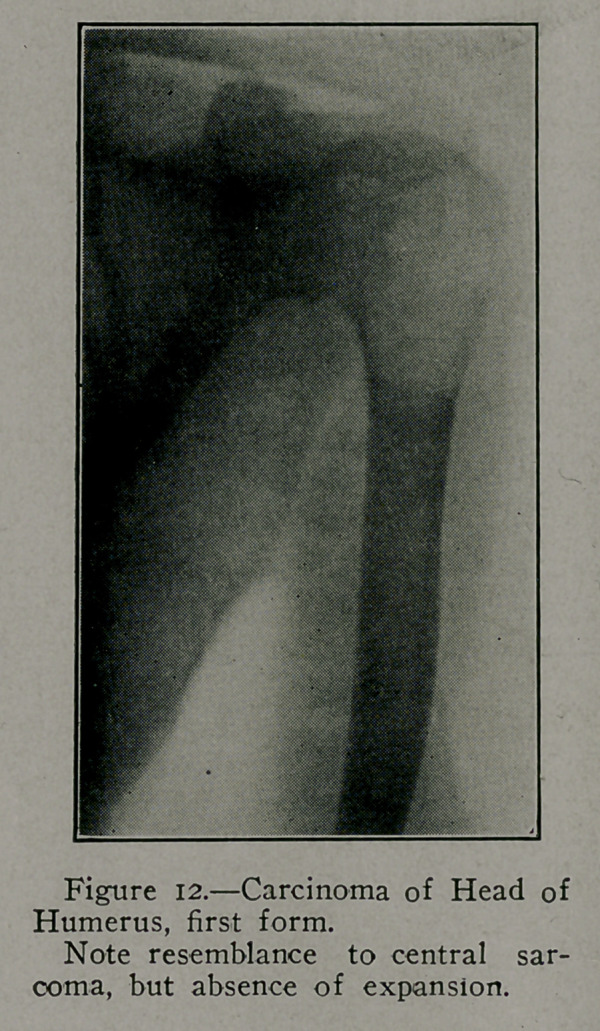


**Figure 13. f13:**
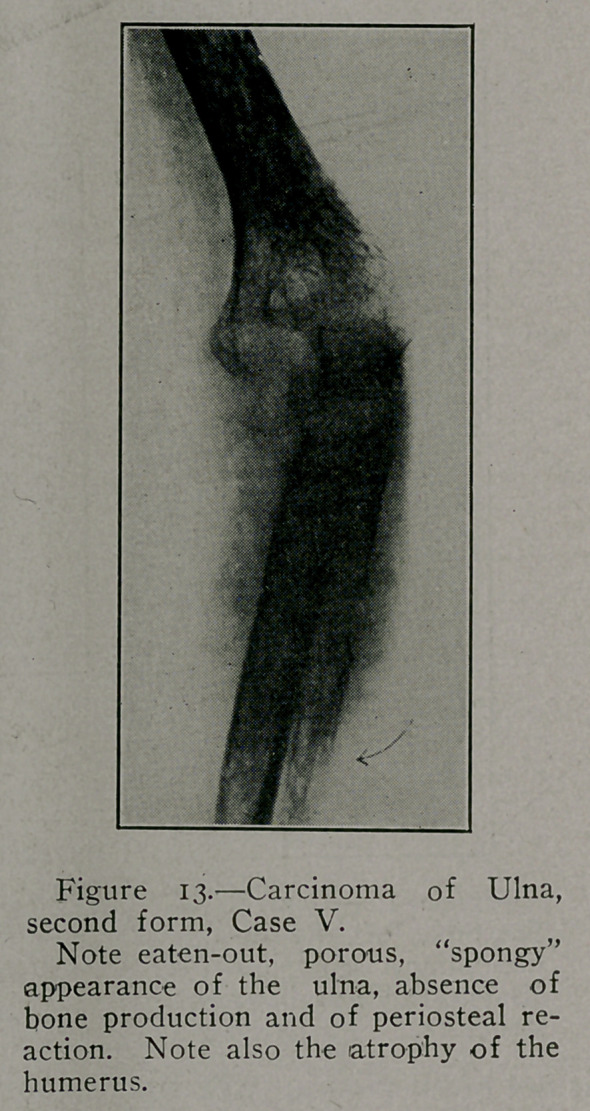


**Figure 14. f14:**
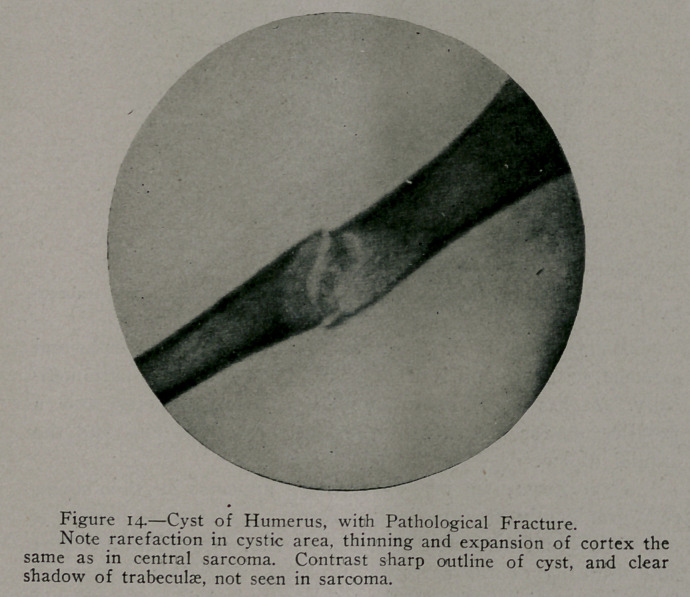


**Figure 15. f15:**
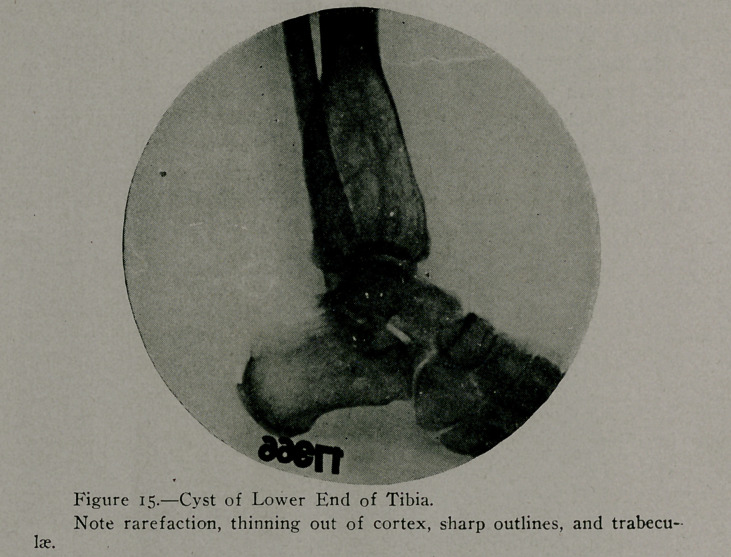


**Figure 16. f16:**
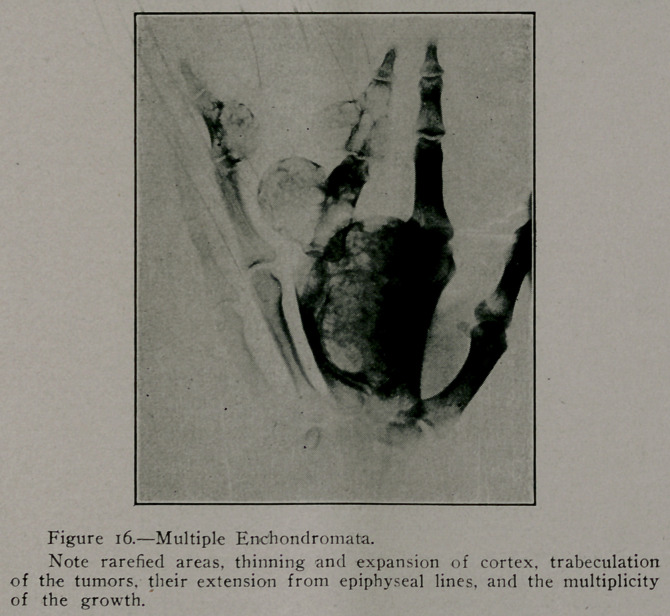


**Figure 17. f17:**
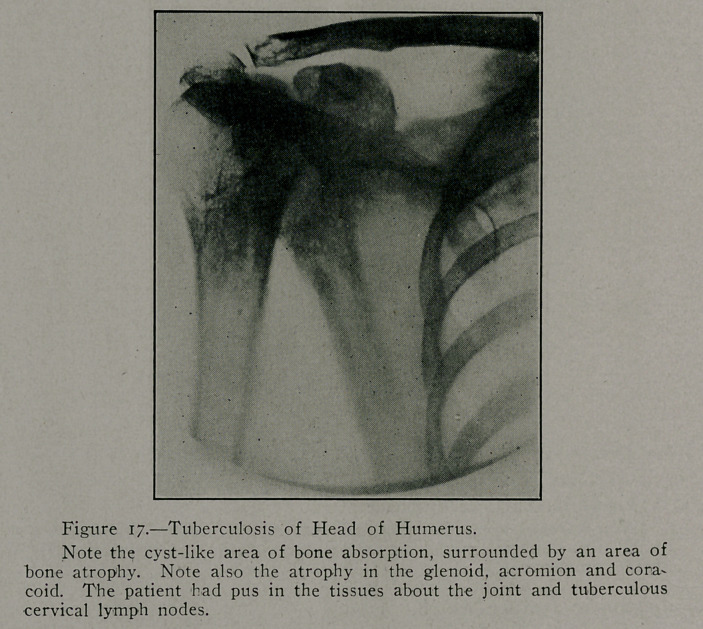


**Figure 18. f18:**